# Clinical features and risk factors analysis for poor outcomes of severe community-acquired pneumonia in children: a nomogram prediction model

**DOI:** 10.3389/fped.2023.1194186

**Published:** 2023-09-21

**Authors:** Changjing Xu, Xuemei Tao, Junlong Zhu, Chao Hou, Yujie Liu, Liya Fu, Wanlong Zhu, Xuping Yang, Yilan Huang

**Affiliations:** ^1^ Department of Pharmacy, The Affiliated Hospital of Southwest Medical University, Luzhou, China; ^2^ Department of Vascular Surgery, The Affiliated Hospital of Southwest Medical University, Luzhou, China; ^3^ Department of Ultrasound, The Affiliated Hospital of Southwest Medical University, Luzhou, China; ^4^ Department of Geriatric Medicine, The Affiliated Hospital of Southwest Medical University, Luzhou, China; ^5^ School of Pharmacy, Southwest Medical University, Luzhou, China

**Keywords:** severe community-acquired pneumonia, children, risk factor, nomogram, predictive model

## Abstract

**Background:**

Pneumonia remains the leading cause of death among children aged 1–59 months. The early prediction of poor outcomes (PO) is of critical concern. This study aimed to explore the risk factors relating to PO in severe community-acquired pneumonia (SCAP) and build a PO-predictive nomogram model for children with SCAP.

**Methods:**

We retrospectively identified 300 Chinese pediatric patients diagnosed with SCAP who were hospitalized in the Affiliated Hospital of Southwest Medical University from August 1, 2018, to October 31, 2021. Children were divided into the PO and the non-PO groups. The occurrence of PO was designated as the dependent variable. Univariate and multivariate logistic regression analyses were used to identify the risk factors of PO. A nomogram model was constructed from the multivariate logistic regression analysis and internally validated for model discrimination and calibration. The performance of the nomogram was estimated using the concordance index (C-index).

**Results:**

According to the efficacy evaluation criteria, 56 of 300 children demonstrated PO. The multivariate logistic regression analysis resulted in the following independent risk factors for PO: co-morbidity (OR: 8.032, 95% CI: 3.556–18.140, *P *< 0.0001), requiring invasive mechanical ventilation (IMV) (OR: 7.081, 95% CI: 2.250–22.282, *P *= 0.001), and ALB* *< 35 g/L (OR: 3.203*,* 95% CI: 1.151–8.912, *P *= 0.026). Results of the internal validation confirmed that the model provided good discrimination (concordance index [C-index], 0.876 [95% CI: 0.828–0.925]). The calibration plots in the nomogram model were of high quality.

**Conclusion:**

The nomogram facilitated accurate prediction of PO in children diagnosed with SCAP and could be helpful for clinical decision-making.

## 1. Introduction

Community-acquired pneumonia (CAP) is the most common cause of hospitalization and a common cause of mortality in children, especially among those under 5 years of age (1). In 2017, The World Health Organization estimated that CAP resulted in more than 800,000 deaths under in children 5 years of age, which accounted for 15% of all deaths in this age group, with most of these deaths occurring in developing countries (2). Early prediction of poor outcomes (PO) is essential for improving the long-term prognosis in children diagnosed with severe community-acquired pneumonia (SCAP). The use of appropriate tools for classifying children with pneumonia and predicting patient outcomes can optimize management (3). A few studies of American children have reported on the use of clinical feature-based predictive statistical models to stratify children with CAP (4, 5). The Respiratory Index of Severity in Children (RISC) and the Pediatric Etiology Research for Child Health (PERCH) score have been used to predict mortality in countries with high pneumonia mortality rates, such as South Africa (6, 7). To date, a model for predicting the clinical outcomes of children with CAP in higher-resourced countries, especially developing countries, has not yet been reported.

Among the predictive statistical models, nomograms are recognized to provide risk estimations with high accuracy, which enable clinicians to standardize clinical decision-making (8, 9). To our knowledge, a nomogram model for predicting the risk of PO in children with SCAP is not available. Hence, this study aimed to establish a nomogram model for the risk prediction of PO in children with SCAP. The use of this model for the evaluation of children with SCAP may enhance our clinical decision-making process, and ultimately, improve patient outcomes.

## 2. Methods

### 2.1. Study subjects

This retrospective case-control study focused on children with SCAP, aged 1–59 months, who were diagnosed in the Department of Pediatrics of Affiliated Hospital of Southwest Medical University from August 2018 to October 2021. All patient clinical data were obtained from the hospital information system.

Pneumonia was diagnosed based on clinical signs or symptoms of acute infection (e.g., fever) and/or acute respiratory illness (e.g., cough), as well as radiographs (e.g., chest x-ray and/or chest CT) (10). CAP was defined as pneumonia diagnosed within 48 h of hospital admission. This is in contrast to hospital-acquired pneumonia (HAP) which was defined as pneumonia that developed 48 h after hospital admission (11). Children with CAP were admitted to the intensive care unit (ICU) or a unit with continuous cardiorespiratory monitoring if they (1) required invasive ventilation via a nonpermanent artificial airway; (2) acutely required the use of noninvasive positive pressure ventilation; (3) were at risk of respiratory failure; (4) demonstrated sustained tachycardia, inadequate blood pressure, or the need for pharmacologic management of their blood pressure or perfusion; (5) showed low oxygen saturation based on pulse oximetry (≦92% with inspired oxygen of ≧0.50); or (6) developed altered mental status. Children with SCAP were defined as children with CAP who were admitted to ICU or a unit with continuous cardiorespiratory monitoring (12).

The following inclusion criteria were met by all children in this study: (1) diagnosed with SCAP; (2) aged 1–59 months; and (3) complete medical records available. Patients were excluded if (1) diagnosed with HAP; (2) experienced non-infectious pneumonitis, such as aspiration, uremic, or hypersensitivity pneumonitis; (3) cases with a history of recent hospitalization; (4) cases with severe immunosuppression; (5) cases with incomplete data. The children were divided into PO and non-PO groups. Children with SCAP who died and whose respiratory tract signs and symptoms of infection (such as fever, tachypnea, nasal flaring, cyanosis, pulmonary rales, etc) did not improve or worsen at the time of discharge were included in the PO group.

The study was approved by the ethics committee of the Affiliated Hospital of Southwest Medical University (No. KY2022004). Informed consent was not required for the retrospective analysis of patient data.

### 2.2. Data collection

Predictors were selected based on clinical expertise and an extensive review of the literature. A pre-designed form was created for data collection. Variables that may affect patient prognosis were included. Clinical and laboratory data such as history, vital signs, physical examination findings, procalcitonin, C-reaction protein, white blood cells count, invasive mechanical ventilation (IMV), and respiratory failure (RF; RF was defined by a partial pressure of arterial O_2_ (PaO_2_) that was <60 mmHg in room air at sea level or an oxygenation index (OI) <300), as well as radiological data, such as chest x-ray and/or chest CT, were collected (Table 1). Co-morbidities including congenital heart disease (e.g., tetralogy of Fallot), underlying pulmonary disease (e.g., bronchopulmonary dysplasia), congenital immunodeficiency, underlying brain disease (e.g., epilepsy), and esophageal atresia were also documented. All predictors were assessed at the time of admission.

**Table 1:** Characteristics of the study population and results of univariate analysis.

Variables	*n*(%), Overall, *n* = 300	PO, *n*(%), *n* = 56	Non-PO, *n*(%), *n* = 244	*χ* ^2^	*p*-value
Age <1 year	193 (64.33)	32 (57.14)	161 (65.98)	1.19	0.275
Male sex	181 (60.33)	28 (50.00)	153 (62.70)	2.56	0.109
Cyanosis^a^	124 (41.33)	34 (60.71)	90 (36.89)	10.67	0.002
Septic shock^a^	20 (6.67)	11 (19.64)	9 (3.69)	16.16	<0.0001
Temperature	38 (12.67)	11 (19.64)	27 (11.07)	2.30	0.129
pH < 7.35^a^	44 (14.67)	23 (41.07)	21 (8.61)	35.81	<0.0001
Malnutrition^a^	37 (12.33)	18 (32.14)	19 (7.79)	22.79	<0.0001
RF^a^	79 (26.33)	30 (53.57)	49 (20.08)	24.64	<0.0001
Chest indrawing	256 (85.33)	45 (80.36)	211 (86.48)	0.913	0.338
AMS^a^	127 (42.33)	40 (71.43)	87 (35.66)	22.43	<0.0001
comorbidity^a^	102 (34.00)	43 (76.79)	59 (24.18)	53.85	<0.0001
Tachypnea	54 (18.00)	15 (26.79)	39 (15.98)	2.91	0.088
Wheezing	147 (49.00)	17 (30.36)	130 (53.28)	2.680	0.208
Grunting	46 (15.33)	12 (21.43)	34 (13.93)	1.435	0.231
Nodal breathing	132 (44.00)	21 (37.50)	111 (45.49)	0.879	0.349
Nasal flaring	79 (26.33)	11 (19.64)	68 (27.87)	1.193	0.273
Hydrothorax	40 (13.33)	11 (19.64)	29 (11.89)	1.748	0.186
Hb < 90 g/L^a^	39 (13.00)	14 (25.00)	25 (10.25)	7.510	0.006
WBC < 4 or >10* 10^9^/L	182 (60.67)	38 (67.86)	145 (59.43)	1.030	0.310
CRP > 20 mg/L	83 (27.67)	17 (30.36)	66 (27.05)	0.11	0.739
PCT > 0.3 ng/ml	117 (39.00)	27 (48.21)	110 (45.08)	0.08	0.783
CK-MB > 4.87 μg/L	129 (43.00)	28 (50.00)	101 (41.39)	1.048	0.306
ALB < 35 g/L^a^	37 (12.33)	17 (30.36)	20 (8.20)	18.69	<0.0001
ALT > 40 U/L	66 (22.00)	18 (32.14)	51(20.90)	2.646	0.104
IMV^a^	42(14.00)	25(44.64)	17(6.97)	50.61	<0.0001

Data are presented as No. (%) unless otherwise indicated.

Temperature, body temperature >41°C or temperature greater than 39°C for more than 5 days; RF, respiratory failure; AMS, altered mental status; Hb, Hemoglobin; WBC, white blood cell count; CRP, C-reactive protein; PCT, procalcitonin; CK-MB, Creatine kinase isoenzyme; ALB, albumin; ALT, Alanine transaminase; IMV, invasive mechanical ventilation; pH, blood pH.

^a^ *p* < 0.05.

Microbiological tests were also performed. Bacterial cultures from blood, sputum, bronchoalveolar lavage fluid, and hydrothorax were conducted. Immunoglobulin M (IgM) serology tests of 9 common respiratory pathogens (*Chlamydophila pneumoniae, Mycoplasma pneumoniae, Legionella pneumophila, influenza A virus, influenza B virus, parainfluenza virus, respiratory syncytial virus, adenovirus, and Rickettsia*).

### 2.3. Statistical analysis

Analyses were conducted using R software (V.4.1.2). Categorical variables were presented as frequency and percentage. The *χ*2 test or Fisher exact test was used to compare the two patient groups. Continuous variables were presented as median with interquartile range (IQR) or standard deviation (SD) and tested using the Mann–Whitney *U*-test. The occurrence of PO was designed as the dependent variable. Univariate and multivariate logistic regression analyses were performed to determine the risk factors influencing the occurrence of PO in patients with SCAP. Independent variables were not included in the model, especially if inconsistent with previous research results and clinical practice.

A nomogram was constructed based on the results of the multivariate analysis. The nomogram set the corresponding score value The nomogram utilized the regression coefficient of each variable in the multivariate logistic regression equation, which is representative of the contribution of each influencing factor to the efficacy, to set the corresponding score for each influencing factor. Then, the sum of the score of each influencing factor was used to calculate the total score.

The effect of individual predictors on PO was reported by using the odds ratios (OR) and the corresponding 95% confidence intervals (95% CI). The performance of the nomogram was estimated by the concordance index (C-index), which is analogous to the commonly reported area under the receiver operating characteristic (ROC) curve. A C-index of 1 indicated perfect concordance. The C-index of most models is 0.7–0.85 (9). Finally, we graphically assessed the agreement between the predicted and observed outcome frequencies (calibration) by using an internal bootstrap validation (1,000 replications with replacement). A two-tailed *p*-value < 0.05 was considered statistically significant.

## 3. Results

### 3.1. Participant characteristics

In total, 429 children with SCAP were diagnosed from August 2018 to October 2021. Of these, 42 were older than 59 months, and 87 had incomplete data. The final study population included 300 children with SCAP, with PO in 56 patients. The median age was 6.83 months [interquartile range (IQR), 2.1–19]. There were 181 (60.33%) boys. Moreover, 102 (34%) children had greater than or equal to 1 co-morbidity (Table 1). Congenital heart disease [65 children (21.67%)] was the most common co-morbidity, followed by pulmonary disease. Furthermore, 42 children (14%) received IMV during hospitalization.

### 3.2. Composition of the pathogens

The *influenza* virus was detected in 2.3% of children and *Mycoplasma pneumoniae* in 6%. A total of 112 strains of pathogenic bacteria were detected from sputum, blood, bronchoalveolar lavage fluid and pleural effusion. *Streptococcus pneumoniae* was the dominant bacterium. There were also 26 strains of *Streptococcus pneumoniae*, 17 strains of *Staphylococcus aureus*, 10 strains of *Escherichia coli*, 10 strains of *Klebsiella pneumoniae*, 9 strains of *Haemophilus influenzae*, 8 strains of *Moraxella catarrhalis,* and 32 strains of other uncommon bacteria identified.

### 3.3. Univariate analysis of PO in patients with SCAP

Table 1 listed all the variables used in the univariate analysis. Results from the univariate analysis showed that the following variables were not significantly different between the PO and non-PO groups: age <1 year, gender, temperature, tachypnea, wheezing, chest indrawing, grunting, nodal breathing, and nasal flaring (Table 1). Compared with the non-PO group, patients with PO had a higher risk of cyanosis, septic shock, malnutrition, altered mental status (AMS), comorbidity, respiratory failure (RF), and the need for IMV (*p *<* *0.05). Laboratory data between the two groups did not demonstrate a statistically significant difference for procalcitonin (PCT), C-reaction protein (CRP), WBC count, creatine kinase-MB (CK-MB), and alanine aminotransferase (ALT) (Table 1). In contrast, hemoglobin, albumin (ALB), and blood pH were significantly lower in the PO group than the non-PO group (*p *<* *0.05). Additionally, the proportion of hydrothorax did not differ significantly between the two groups (*p* > 0.05).

### 3.4. Multivariate regression analysis of PO in patients with SCAP

Significant variables from the univariate analysis were considered as the independent variables. PO was designated as the dependent variable. These variables were included in the multivariate logistic regression analysis (Table 2). The results showed that comorbidity (OR: 8.032, 95% CI: 3.556–18.140, *P *< 0.0001), the need for IMV (OR: 7.081, 95% CI: 2.250–22.282, *P *= 0.001), and ALB* *<* *35 g/L (OR: 3.203*,* 95% CI: 1.151–8.912, *P *= 0.026) were independent risk factors of PO in children with SCAP.

**Table 2:** The results of multivariate logistic regression analysis.

Factors	*p*-value	OR	OR(95%CI)	
			Lower limit	Upper limit
Cyanosis	0.657	0.831	0.444	2.382
Septic shock	0.946	0.964	0.280	3.571
Malnutrition	0.200	1.833	0.725	4.635
AMS	0.190	1.799	0.748	4.328
Comorbidity^a^	<0.0001	8.032	3.556	18.140
RF	0.537	0.730	0.269	1.982
Hemoglobin < 90 g/L	0.410	0.635	0.216	1.870
ALB < 35 g/L^a^	0.026	3.203	1.151	8.912
pH < 7.35	0.117	2.221	0.818	6.033
IMV^a^	0.001	7.081	2.250	22.282

AMS, altered mental status; RF, respiratory failure; ALB, albumin; pH, blood pH; IMV, invasive mechanical ventilation.

^a^ *p* < 0.05.

### 3.5. Nomogram construction and validation

**Figure 1:**
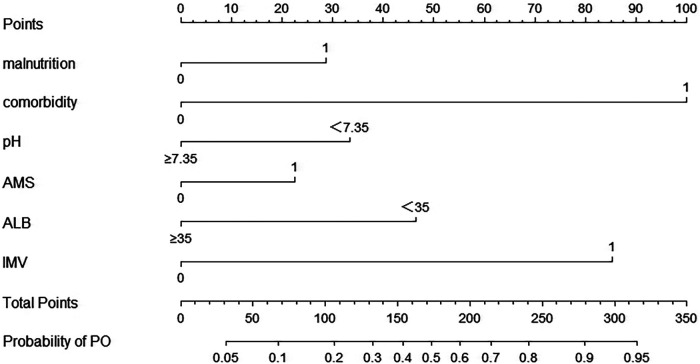
The nomogram for calculating the risk score and predicting the risk of PO in SCAP patients. To calculate total score and predicted probability of PO, points from individual variables are added and a vertical line is drawn from the total points line at the bottom downward to determine the predicted probability of PO due to SCAP. PO, poor outcome; SCAP, severe community-acquired pneumonia; pH, blood pH; AMS, altered mental status; ALB, albumin; IMV, invasive mechanical ventilation.

Factors, such as cyanosis, RF, septic shock, and hemoglobin <90 g/L, and factors with an OR of <1 which were inconsistent with clinical practice, were not included in the prediction model. The remaining variables (malnutrition, AMS, comorbidity, ALB* *<* *35 g/L, pH* *<* *7.35, and IMV) from multivariate logistical regression analysis were used to establish a nomogram for the risk of PO (Figure 1). The nomogram was generated by assigning a weighted score to each influencing factor. The highest score was 350 points, and the range of PO incidence was 0.05–0.95. A higher score calculated from the sum of the distribution points of each high-risk factor in the nomogram corresponded to a higher risk of PO.

**Figure 2:**
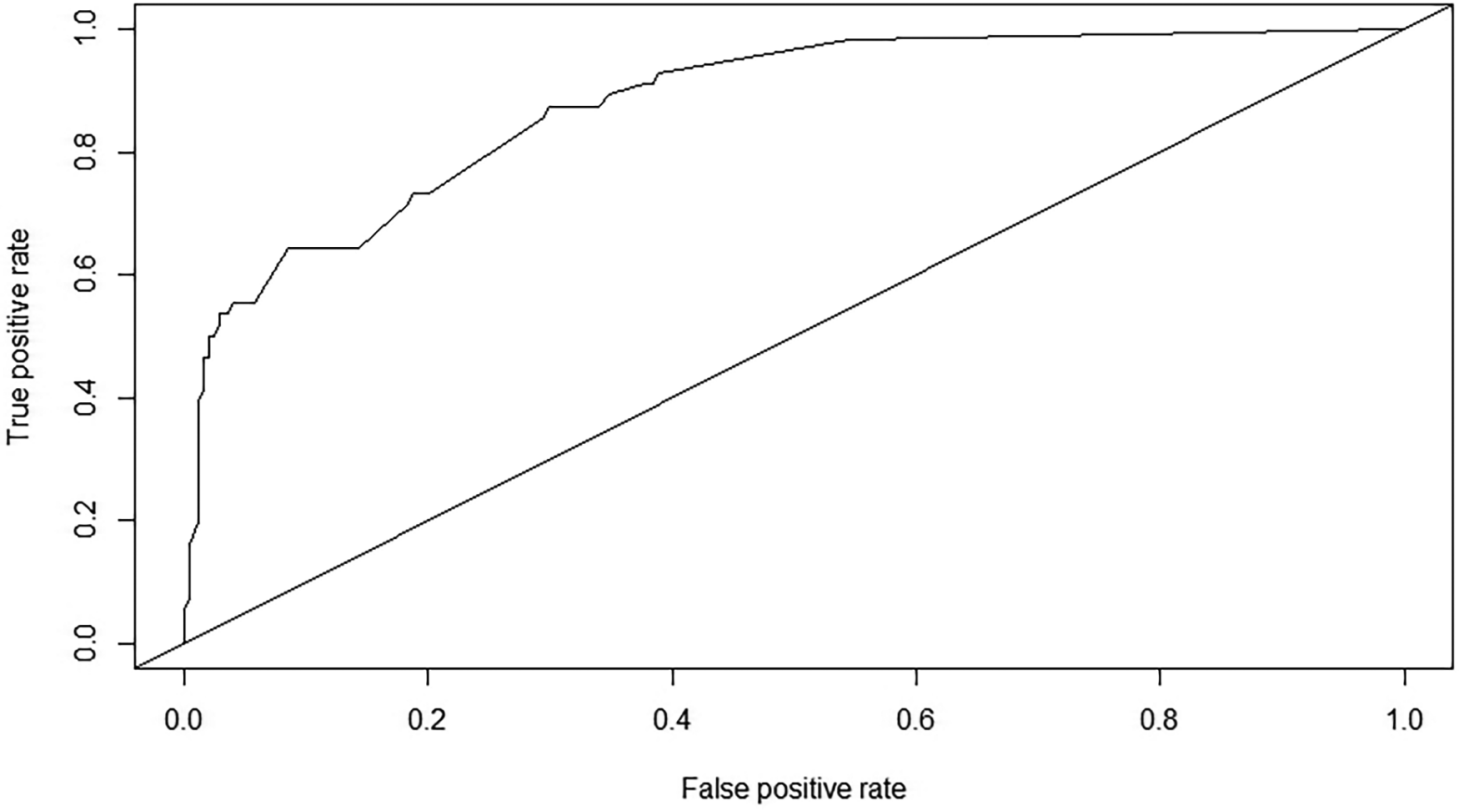
ROC curve and AUC of the nomogram, AUC = 0.876 (95% CI: 0.828–0.925).

**Figure 3:**
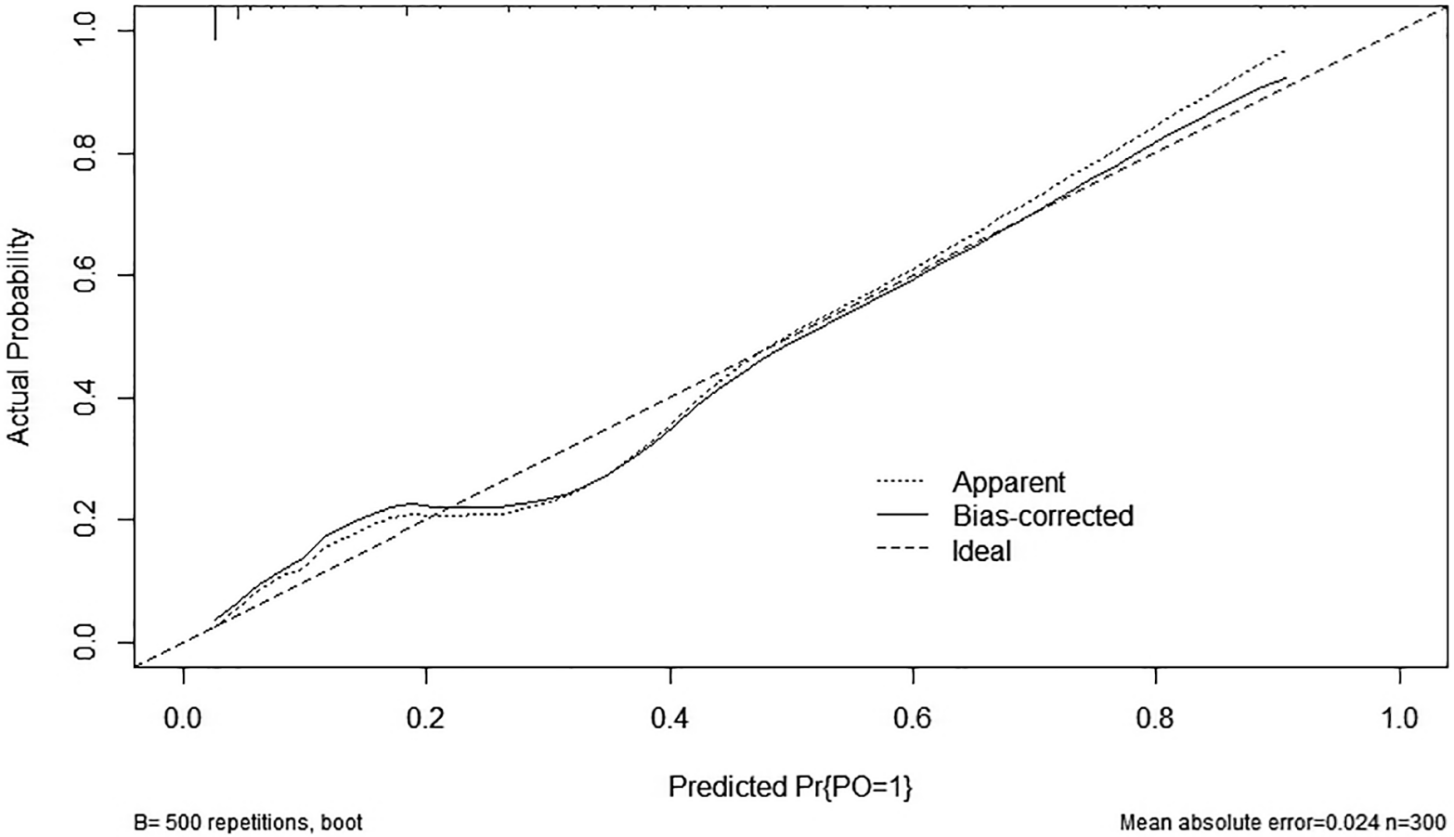
The calibration plot of the nomogram. The horizontal axis indicates the risk of PO occurrence predicted by the nomogram, and the vertical axis represents the actual observed risk of PO occurrence.

The resulting model was internally validated using the bootstrap validation method. The nomogram demonstrated good performance in estimating the risk of PO, with a C-index of 0.876 (95% CI: 0.828–0.925). The area under the ROC curve was 0.876 (Figure 2). Importantly, the calibration chart showed that the nomogram had a sufficient degree of fit for predicting the incidence of PO in children with SCAP (Figure 3).

## 4. Discussion

CAP is the most common infectious disease during childhood, Nevertheless, no validated nomogram model exists to predict the risk of PO caused by SCAP. Although vaccination has significantly reduced the incidence and mortality of severe pneumonia in children in recent years, vaccination is not universally available. Pneumonia remains the leading cause of death among children, aged 1 month to 5 years (13). There are limitations in the prognostic evaluation of pneumonia among children via a single symptom or sign. At present, precise, comprehensive, and practical evaluation standards are lacking. Additionally, an adequate instrument to predict patient outcomes is not available (3). Previous studies have explored the risk factors associated with poor clinical outcomes of SCAP. However, a nomogram based on of multivariate logistic regression analysis results was not included (14). In this study, univariate and multivariate logistic regression analyses were used to identify the risk factors for PO and a PO-predictive nomogram was constructed to comprehensively and accurately estimate the risk of PO for each patient with SCAP.

This study showed that SCAP mainly occurred in infancy, which is consistent with that of Walker et al. Walker et al. reported that the incidence and mortality of SCAP were highest in children aged less than 1 year (15). The established cause was suggested to be related to a vulnerable and underdeveloped respiratory system in this age group, which led to greater infection susceptibility. Gender distribution in our study revealed a male predominance (1.5:1). Previous studies also showed similar results (12, 16). The literature suggested that viruses were most commonly detected in children with pneumonia, especially *respiratory syncytial virus* (4, 17)*.* In the present study, bacteria were the most common pathogens. This study did not collect nasopharyngeal aspirates or swabs for the detection of common respiratory virus antigens.

To accurately predict the risk of PO in children with SCAP, we identified 6 factors using univariate and multivariate analyses, namely, malnutrition, comorbidity, blood pH* *<* *7.35, AMS, ALB* *<* *35 g/L, and the need for IMV. These factors were used to establish a nomogram for the risk of PO. To the best of our knowledge, this is the first nomogram study of the risk of PO caused by SCAP. Based on the AUC and calibration curve evaluations, the nomogram model showed good accuracy and consistency. Thus, the nomogram model can effectively contribute to the early prediction of PO in children with SCAP to facilitate timely therapeutic and preventive measures. To a certain extent, the prognosis of children with SCAP may also be improved.

This study showed that co-morbidity, ALB* *<* *35 g/L, and the need for IMV were independent risk factors for PO in children with SCAP. In this study, congenital heart disease was the most common co-morbidity in children with SCAP. Children with congenital heart disease are more likely to develop repeated respiratory tract infections (18), that require hospitalization and antimicrobial application. These risk factors of pneumonia are potentially related to multi-drug-resistant bacteria, which also increases mortality (19). Previous studies have also confirmed that basic cardiopulmonary diseases including congenital heart disease, bronchopulmonary dysplasia, cystic fibrosis, asthma, and other systemic diseases, can induce and aggravate pneumonia (20, 21). Koh et al. also showed that comorbidity was an independent risk factor for PO in patients with SCAP (16). Therefore, the treating team should pay more attention to comorbidities, treat the comorbidities accordingly, and be vigilant in monitoring outcomes. Moreover, Koh et al. reported that IMV increased the risk of ventilator-associated pneumonia (VAP), which was in turn, associated with PO (16). Furthermore, children undergoing IMV often have severe hypoxemia. A previous study showed that the severity of hypoxemia was an independent risk factor of mortality among patients (22). Conversely, patients may also experience other complications, such as VAP, pulmonary barotrauma, laryngeal edema, airway injury, and pneumothorax (23), and these complications may also adversely affect outcomes in severe cases. For children receiving IMV, the medical teams should aim to reduce the above-mentioned complications by using various means, e.g., manual lung inflation, vibration expectoration, and early functional exercise (24). Another study showed that serum ALB can rapidly decrease in acute infection (25). Furthermore, the reduction of ALB was closely related to increased pulmonary vascular permeability, which has important clinical value in predicting the severity and prognosis of pneumonia (26, 27).

Metabolic acidosis is very common in septic patients, who are often critically ill, and the severity of metabolic acidosis is associated with poor clinical outcomes (28, 29). In the current study, a blood pH of <7.35 was used as a key indicator of metabolic acidosis. The PO group demonstrated a higher tendency for a blood pH of <7.35 than the non-PO group. Jroundi et al. (30) reported that cyanosis is an independent risk factor for adverse outcomes among children with severe pneumonia in middle-income countries. However, in our study, the variable was significantly different between the two groups only in the univariate analysis. Differences in the patient population and study design may account for these differences.

Nomogram is used to predict the probability of a clinical event occurring in an individual. This has become more common with more rapid computation through user-friendly digital interfaces and increased accuracy (9). This is the first study to create a simple visual prediction nomogram model for predicting PO in patients with SCAP using six factors. The C-index was used to assess the concordance, which was graded as very good (0.80–1.00) (31). Our nomogram also showed good performance for prediction (C-index of 0.876), which may improve over time and form the basis of new ideas for the early identification and intervention of PO in patients with SCAP.

Lung function parameters play a pivotal role in the assessment of respiratory diseases (32). Early lung infections are negatively associated with most lung function parameters (33). For children with SCAP, tests of lung function are helpful for assessing disease severity and predicting poor outcomes. The prediction model will be more objective and convincing by adding lung function to risk factors analysis. However, this was a retrospective study and lung function tests were not done routinely in SCAP children at our center. This will be further evaluated in our prospective study.

This study has some limitations. First, this retrospective study was based on reviewing medical records from a single institution and the sample size was small. Second, bootstrap repeated sampling was used to verify the model in the study. Finally, we didn’t add some factors (e.g., lung function test) that may be meaningful to risk factors analysis due to missing data. It is necessary to validate our results with those of other centers. In the future, a prospective multi-center in-sample study to further confirm the reliability of the nomogram is warranted.

## 5. Conclusions

A prognostic nomogram for predicting the risk of poor outcomes in children with SCAP was constructed. The nomogram demonstrated good discrimination and calibration in our study. The nomogram may be used to predict patient outcomes as early as at the time of admission, which may contribute to improving the clinical management of SCAP.

## Data Availability

The original contributions presented in the study are included in the article/Supplementary Material, further inquiries can be directed to the corresponding authors.
